# Relationship between Memory Load and Listening Demands in Age-Related Hearing Impairment

**DOI:** 10.1155/2021/8840452

**Published:** 2021-06-04

**Authors:** Julia Pauquet, Christiane M. Thiel, Christian Mathys, Stephanie Rosemann

**Affiliations:** ^1^Biological Psychology, Department of Psychology, School of Medicine and Health Sciences, Carl von Ossietzky Universität, 26111 Oldenburg, Germany; ^2^Cluster of Excellence “Hearing4all”, Carl von Ossietzky Universität Oldenburg, 26111 Oldenburg, Germany; ^3^Institute of Radiology and Neuroradiology, Evangelisches Krankenhaus, Carl von Ossietzky Universität Oldenburg, 26122 Oldenburg, Germany; ^4^Research Center Neurosensory Science, Carl von Ossietzky Universität Oldenburg, 26111 Oldenburg, Germany

## Abstract

Age-related hearing loss has been associated with increased recruitment of frontal brain areas during speech perception to compensate for the decline in auditory input. This additional recruitment may bind resources otherwise needed for understanding speech. However, it is unknown how increased demands on listening interact with increasing cognitive demands when processing speech in age-related hearing loss. The current study used a full-sentence working memory task manipulating demands on working memory and listening and studied untreated mild to moderate hard of hearing (*n* = 20) and normal-hearing age-matched participants (*n* = 19) with functional MRI. On the behavioral level, we found a significant interaction of memory load and listening condition; this was, however, similar for both groups. Under low, but not high memory load, listening condition significantly influenced task performance. Similarly, under easy but not difficult listening conditions, memory load had a significant effect on task performance. On the neural level, as measured by the BOLD response, we found increased responses under high compared to low memory load conditions in the left supramarginal gyrus, left middle frontal gyrus, and left supplementary motor cortex regardless of hearing ability. Furthermore, we found increased responses in the bilateral superior temporal gyri under easy compared to difficult listening conditions. We found no group differences nor interactions of group with memory load or listening condition. This suggests that memory load and listening condition interacted on a behavioral level, however, only the increased memory load was reflected in increased BOLD responses in frontal and parietal brain regions. Hence, when evaluating listening abilities in elderly participants, memory load should be considered as it might interfere with the assessed performance. We could not find any further evidence that BOLD responses for the different memory and listening conditions are affected by mild to moderate age-related hearing loss.

## 1. Introduction

Age-related hearing loss (presbyacusis) affects a large part of the older population and encompasses the decline of hearing ability in higher frequencies [1, 2]. Alongside auditory constraints, presbyacusis has been associated with a decline of cognitive capabilities [3], especially in working memory performance [4, 5] as well as with lower quality of life [2] and depression [6]. Individuals affected by presbyacusis particularly report problems when listening to speech-in-noise, as this requires expending more effort to understand and process speech [7].

This listening effort may bind cognitive resources that might otherwise be available for concurrent cognitive requirements when processing spoken language. Even in young, normal-hearing listeners, it was shown that imposing additional memory load on a sentence intelligibility task changed the pupil dilation response, which can be an indicator of listening effort [8]. Further, the working memory span was significantly worse when words were presented under difficult—but still audible—listening conditions [9, 10]. For older hard of hearing adults, those with a larger working memory capacity rated listening in noise as less effortful than hard of hearing adults with a lower working memory capacity, indicating that effortful listening is closely linked to verbal working memory [11]. Another study found that increased levels of stimulus complexity requiring higher memory load decreased auditory accuracy in a recall task in middle-aged and older normal-hearing adults [12]. This research shows that demands on listening and working memory may interact with each other, which is especially relevant for presbyacusis.

Adverse listening conditions require people to use more resources which result in the recruitment of additional brain areas, primarily in the frontal cortex. Several studies found increased neural responses in areas including the cingulo-opercular network, the inferior frontal cortex, the insula, and the middle frontal gyrus during difficult listening conditions [13–19]. Similarly, high compared to low verbal working memory load leads to increased dorsolateral and ventrolateral prefrontal cortex activation [20]. This increased activity may interfere with the recruitment of frontal areas in adverse listening conditions. However, whether such interference occurs in the prefrontal cortex and whether it is increased in the hard of hearing has not been tested.

Hence, the aim of this study was to investigate how increased demands on working memory interact with increased demands on listening effort in hard of hearing and normal-hearing participants. Subjects with mild to moderate untreated age-related hearing impairment and age-matched participants with normal hearing were studied with functional magnetic resonance imaging (fMRI). The main fMRI task was a verbal working memory task using full sentences. Here, we employed a 2 × 2 design with varying memory load (high or low) and listening condition (difficult or easy). Participants were asked to listen to two sentences and either recognize a word from the last sentence (low load) or to compare whether a noun or a verb was the same in both sentences (high load). This allowed for a rather naturalistic setting to measure working memory, as information had to be extracted from full-length sentences. We expected larger drops of performance in hard of hearing participants under either high memory load or difficult listening conditions, meaning that the performance difference between easy and difficult listening conditions under high memory load is greater in the hard of hearing as compared to the normal hearing. On the neural level as measured by the BOLD response, we hypothesized (1) stronger engagement of frontal brain areas, including the cingulo-opercular network, the inferior frontal and premotor cortices, the insula, and the middle frontal gyrus, when listening conditions are more difficult [17]; and (2) higher engagement of working memory-related areas, especially the dorsolateral prefrontal and superior parietal cortex, under high memory load conditions [18] in hard of hearing compared to normal-hearing participants.

## 2. Materials and Methods

### 2.1. Participants

**Figure 1:**
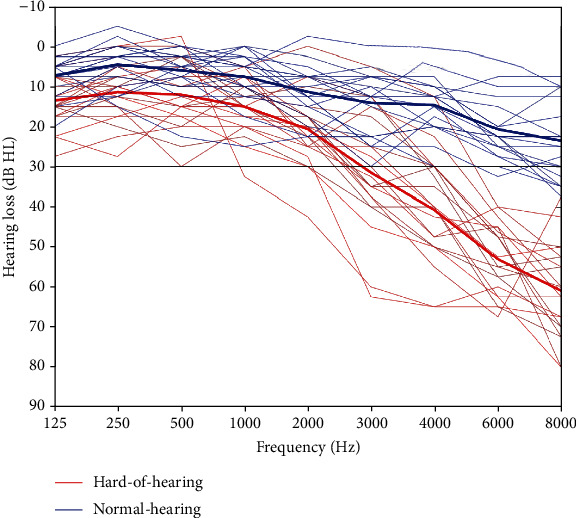
Pure tone audiometry for hard of hearing (red) and normal-hearing (blue) participants. Thin colored lines represent mean over both ears of a participant, and thick colored lines represent the group mean. The black line indicates 30 dB threshold for hearing impairment.

For this study, we recruited two age-matched groups of participants with either age-appropriate hearing abilities or mild to moderate age-related hearing loss. In total, 43 participants between the age of 50 and 75 years took part in this study. Four participants had to be excluded from the analysis due to movement artifacts in the MRI (>3 mm). Audiometric assessment and classification into hard of hearing and normal-hearing participants were executed by trained staff. Normal hearing abilities were classified as a mean value of 25 dB or better in pure tone audiometry in the frequencies from 2000 to 8000 Hz [21] All participants had to have a symmetric hearing in both ears. Of the remaining 39 participants, *n* = 19 did not exceed the threshold of 25 dB and were therefore classified as normal hearing, and *n* = 20 participants were classified as hard of hearing (Figure 1). Participants with low-frequency hearing loss were not included in the study. Hard of hearing and normal-hearing participants were matched based on their age and had a mean age of *M* = 64.9 (SD = 5.67) years and *M* = 63.32 (SD = 6.13) years, respectively. All participants were screened for MRI contraindications. The subjects were right-handed, native German speakers, had normal or corrected-to-normal vision, and had no history of neurological or psychological disease. Furthermore, participants had no experience with hearing aids. The project was approved by the ethics committee of the Carl von Ossietzky University Oldenburg (“Kommission für Forschungsfolgenabschätzung und Ethik”) and adhered to the Declaration of Helsinki [22]. Participants gave their written informed consent to participate in the study and received monetary compensation. This study was preregistered on http://AsPredicted.com/ on the 27th of March 2019 and has the document number 21386.

### 2.2. Verbal Working Memory Task

**Figure 2:**
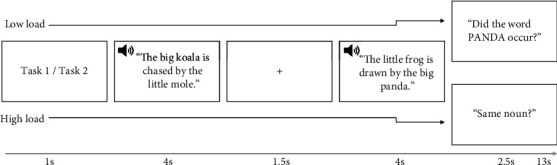
Verbal working memory task. In each trial, two consecutive sentences separated by the presentation of a fixation cross were presented. Beforehand, it was indicated whether participants were supposed to perform task 1, which corresponds to (a) the low memory load condition (i.e., indicate whether a specific word occurred in the last sentence), or task 2, which corresponds to (b) the high memory load condition (i.e., indicate whether the noun/verb was the same in both sentences). Both memory load conditions were presented either at the 80% speech reception threshold (difficult listening condition) or at the 80% threshold +5 dB (easy listening condition).

Within the MRI, participants performed a verbal working memory task. The stimulus material consisted of sentences from the Oldenburg Audiologically and Linguistically Controlled Sentences [23] read by a male speaker. The task was presented in a 2 (memory load: high vs. low, varying task difficulty) by 2 (listening condition: difficult vs. easy, varying the sound intensity level) within-subjects design with an additional rest condition resulting in five conditions. In each trial, participants listened to two consecutive sentences while viewing a fixation cross. In the low memory load condition, participants had to indicate whether a specific word occurred in the second sentence (in 50% of the trials, we asked for a noun, and in 50%, we asked for a verb). In the high memory load condition, participants had to indicate whether any noun (50% of trials) or any verb (50% of trials) was the same in both sentences (see Figure 2). This requires increased load as participants had to keep both sentences in their working memory and compare them for similarities, rather than just recalling a specific word in the second sentence. As such, the high memory load served as a way to evaluate verbal working memory. Responses (yes or no) were given by button presses (left and right thumb, respectively). In addition, sentences were presented at two different sound intensity levels based on the participant's speech reception threshold (SRT) as measured by the Oldenburger Sentence Test (OLSA) inside the scanner [24]. Sentences in the difficult listening condition were presented at 80% speech reception threshold (obtained by the OLSA inside the scanner). For sentences in the easy listening condition, 5 dB was added to the sound level required to obtain 80% SRT. An example trial can be found at https://osf.io/rs62e/. The order of the four conditions and the rest condition was randomized once in such a way that no two rest conditions would follow each other. This order was then presented to all participants in the same manner. Each sentence pair was assigned to one condition and would only appear once (e.g., sentence 3 would only appear in condition high memory load/easy listening). Sentences within the conditions were shuffled across trials for each participant. Hence, the order of the conditions was the same for each participant, but the order of the sentences that appeared within a condition was shuffled. Rest conditions lasted 13 seconds and consisted of a white fixation cross on a grey background. Task trials also had a duration of 13 seconds. In total, 84 trials were performed, leading to a task duration of approximately 18 minutes. After the measurement, participants were asked to rate the difficulty of the task conditions on a four-point scale (1 = not difficult; 4 = very difficult).

The task was programmed using Presentation® software (Version 18.0, Neurobehavioral Systems, Inc., Berkeley, CA, http://www.neurobs.com/). The task was presented via a projector (DATAPixx2, VPixx Technologies Inc.) on a screen, which participants could see through a mirror attached to the head coil. Auditory stimulation was achieved via MRI-compatible headphones (Opto Active, Optoacoustics Ltd., Israel) while active noise cancelling was enabled to filter out high-frequency gradient switching noise. Two buttons of MRI-compatible response pads served to record the participants' responses (Current Designs, Philadelphia, PA, USA, http://www.curdes.com/).

### 2.3. Cognitive Tests

In addition, both groups were screened with a set of cognitive tests. To test cognitive flexibility, Trail 1 and Trail 5 of the Comprehensive Trail Making Test (CTMT) [25] were administered. The general cognitive state was assessed using the Montreal Cognitive Assessment (MoCA) [26]. The Stroop color-word interference test [27] was used to determine inhibition control. Participants' executive function was assessed with the Intra-Extradimensional Set Shift task of the Cambridge Neuropsychological Test Automated Battery (CANTAB) [28]. Right-handedness was verified with the Edinburgh Inventory [29]. Depression was evaluated using the Geriatric Depression Scale (GDS) [30]. Participants also filled out a multiple choice word test (WST) [31], a short verbal intelligence test.

### 2.4. Listening Tests

To further characterize our sample, several additional listening tests were administered. To assess their understanding of speech-in-noise, participants completed the OLSA twice, once outside the scanner with speech noise (SNR) and once inside the scanner with MRI noise (dB). Listening effort was captured with the Adaptive Categorical Listening Effort Scaling (ACALES) test [32], an adaptive, computer-based procedure in which participants listen to speech-in-noise and have to rate their listening effort on a 13-point scale. Participants also filled out a questionnaire regarding listening effort in daily life, where they had to indicate their perceived effort of a described daily situation on a scale from 1 to 10 [33].

### 2.5. MRI Data Acquisition

MRI data were acquired using a 3 Tesla Siemens Magnetom Prisma scanner (Siemens AG, Erlangen, Germany) and a 20-channel head coil. Participants wore hearing protection and MRI-compatible active noise-cancelling headphones. The remaining space was filled with foam pads to minimize head movement and to keep the headphones in position. Functional images with a blood oxygen level dependent (BOLD) contrast were created with an echo planar imaging (EPI) sequence (repetition time (TR) = 1800 ms, echo time (TE) = 30 ms, flip angle = 75°, voxel size = 3 mm, slice thickness = 4 mm, number of slices = 33, field of view = 192 × 192 mm). For the verbal working memory task, 635 images were collected. Additionally, a T1-weighted structural image was acquired using a magnetization prepared rapid gradient-echo (MPRAGE) sequence (TR = 2000 ms, TE = 2.07 ms, flip angle = 9°, voxel size = 0.75 mm^3^, number of slices = 224, matrix = 320 × 320, field of view = 240 × 240 mm). Resting state and diffusion tensor imaging data were acquired within a larger project on hearing impairment and are not presented here.

### 2.6. fMRI Data Analysis

MRI data were preprocessed and analyzed statistically using Matlab R2016a (The MathWorks, Inc., Natick, Massachusetts, United States) and SPM12 (Wellcome Trust Centre for Neuroimaging, London, UK). Preprocessing of the functional images included a realignment estimation, coregistration to the structural image, normalization into MNI standard space using parameters obtained during structural image segmentation, and, finally, smoothing using an 8 mm^3^ full-width-at-half-maximum Gaussian kernel. The BOLD response for each participant was modeled with a general linear model (GLM) including the whole length of the 13-second trial. Low-frequency noise was removed with a temporal high-pass filter at 128 seconds. Temporal autocorrelations were corrected using an autoregressive model of the first order. The parameters of the GLM were estimated using a restricted maximum likelihood approach. The four conditions of the verbal working memory task as well as the rest condition were entered into the model as regressors of interest. The six movement parameters obtained during preprocessing were entered as nuisance regressors. On the first level, contrast images for each subject were calculated for each condition (low load/easy listening, low load/difficult listening, high load/easy listening, and high load/difficult listening) against the rest condition. At group level, a full factorial model using the contrast images created on the first level was performed. The two groups were entered as a factor as well as the task conditions of high/low load and easy/difficult listening, yielding a 2 × 2 × 2 model. We analyzed the main effects of memory load, listening condition, and group, as well as interactions on a whole brain level using cluster-based corrections for multiple comparisons (voxel significance threshold of *p* < 0.001; family-wise error correction of *p* < 0.05). Additionally, specific contrasts for memory load (high > low, and vice versa) and for listening condition (easy > difficult, and vice versa) for hard of hearing compared to normal-hearing participants were computed using a working memory mask including dorsolateral prefrontal and superior parietal cortex, and an auditory effort mask including the cingulo-opercular network, the inferior frontal and premotor cortices, the insula, and the middle frontal gyrus. Results were projected onto a surface rendering for better visibility using SPM12's render option and an example surface from SPM12. To provide evidence for the absence of BOLD response differences between groups, we computed an additional Bayesian independent sample *T*-test with default prior option (Cauchy distribution) comparing beta values between both groups. This data analysis was performed in JASP (JASP Team, 2020; Version 0.14.1).

### 2.7. Behavioral Data Analysis

All behavioral data were analyzed using SPSS 25 (IBM Corp, 2017, Armonk, NY: IBM Corp.). Behavioral data were first controlled for normal distribution using a Kolmogorov-Smirnov test. The performance score was entered into an ANOVA with factors group, listening condition, and memory load as independent variables, the same was done for the difficulty ratings for each condition. Sum scores of the MoCa (cognitive abilities), multiple choice word test (verbal intelligence), listening effort questionnaire, and GDS (depression) were evaluated. For executive functioning, we used the Stroop Interference score as well as the sum of errors in the Intra-Extradimensional Set Shift task during within- and between-set switches. Further, the difference in time needed to complete Trail 5 and Trail 1 on the CTMT (cognitive flexibility) was evaluated. For the hearing measures, the mean hearing loss in the frequency range from 2000 to 8000 Hz was used, as well as the SRT measured by the OLSA. In the ACALES, the mean SNR at every rating stage was used. All cognitive and hearing tests were evaluated for group differences using two-sample *t*-tests. When applicable, a Mann–Whitney *U*-test was used instead.

## 3. Results

### 3.1. Cognitive Tests

Normal-hearing and hard of hearing participants did not differ in age, handedness, or cognitive capabilities in any task or subtask. Neither group exhibited depressive symptoms. Only in the verbal intelligence test (WST) normal-hearing participants performed significantly better than hard of hearing participants, *t*(37) = 2.06, *p* = 0.046, and *d* = 0.68. Results for the cognitive tasks CTMT, MoCa, Stroop, WST, and Set Shifting can be found in Table 1.

### 3.2. Listening Tests

Statistical comparison of audiograms confirmed our classification of participants into a normal-hearing and hard of hearing group. Hard of hearing participants had significantly reduced hearing compared to normal-hearing participants in the frequency range from 2000 to 8000 Hz, *t*(37) = −9.91, *p* < 0.001, *d* = −3.25. Hard of hearing participants exhibited a significantly worse speech-in-noise reception outside the scanner (i.e., with speech noise) than normal-hearing participants, *U* = 6, *p* = 0.011; however, the groups did not differ in their threshold when the same test was performed inside the scanner (i.e., with MRI noise). There was no group difference in subjectively experienced daily listening effort. In the ACALES, both groups differed significantly at twelve of thirteen stages of listening effort rating except for the condition of “high effort” (Table 2). For SNRs rated as “low effort,” hard of hearing participants showed lower SNRs than normal-hearing participants, *t*(37) = 2.17, *p* = 0.035, as well as for SNRs rated as “medium effort,” *t*(37) = 2.51, *p* = 0.016 (Table 2). For display purposes, only three rating stages are displayed here. All values for the ACALES can be found in Supplementary Table 1.

### 3.3. Behavioral Data MRI

**Figure 3:**
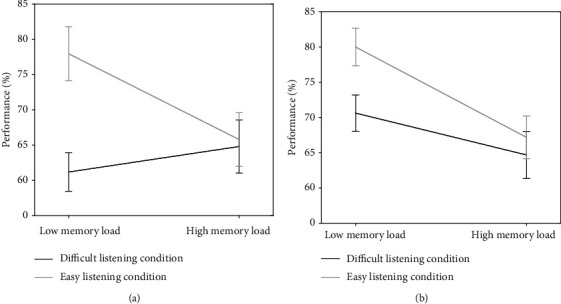
The interaction effect of listening and memory load conditions in (a) hard of hearing and (b) normal-hearing participants. Error bars denote standard error of the mean.

Performance values in the verbal working memory task are displayed in Figure 3 and Supplementary Table 2.

Participants performed significantly worse under high compared to low memory load conditions, (main effect of memory load, *F*(1, 148) = 8.85, *p* = 0.003, *η*^2^ = 0.056) as well as under difficult compared to easy listening conditions (main effect of listening condition, *F*(1, 148) = 10.44, *p* = 0.002, *η*^2^ = 0.066). Additionally, participants' performance was influenced by the listening condition when memory load was low, but not when memory load was high (interaction of listening condition∗memory load, *F*(1, 148) = 6.11, *p* = 0.015, *η*^2^ = 0.040, Figures 3(a) and 3(b)). There was no significant effect of group on the performance and neither the interaction of group∗listening condition, nor group∗memory load nor group∗listening condition∗memory load was significant.

**Figure 4:**
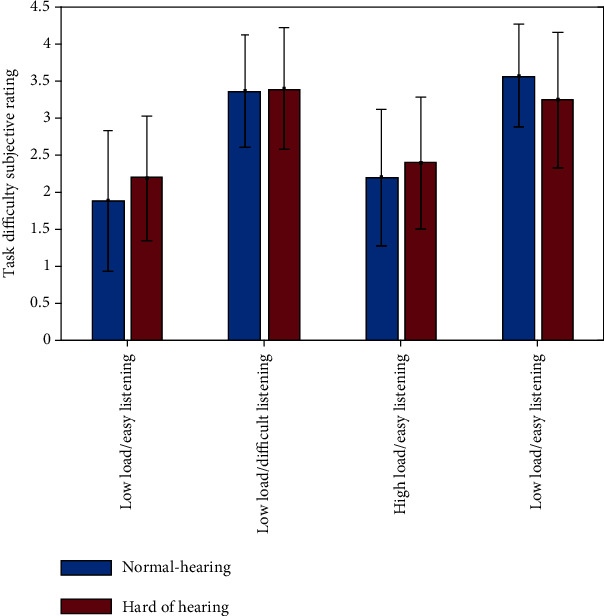
Difficulty ratings of the task conditions. Participants rated each task condition on a scale from 1-4 with 4 being the most difficult. Error bars depict the standard deviation from the mean.

Participants rated the difficult listening condition as significantly more difficult than the easy listening condition (main effect of listening condition, *F*(1, 148) = 80.97, *p* < 0.001, *η*^2^ = 0.354). However, there was neither a difference in the rating of the memory load conditions nor in ratings between the groups (Figure 4).

### 3.4. Neuroimaging Data

**Figure 5:**
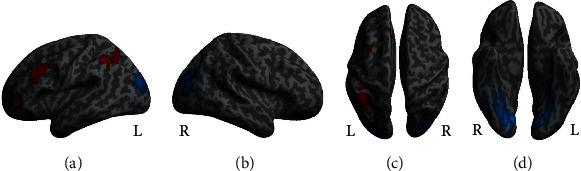
Main effect of the memory load condition. Peak activation sites include the left supramarginal gyrus, the left middle frontal gyrus, and the left supplementary motor cortex (increased BOLD activity under high compared to low memory load, red) as well as left and right lingual gyri and left and right middle occipital gyri (increased BOLD activity under low compared to high memory load, blue). (a) Left hemisphere view, (b) right hemisphere view, (c) top view (left hemisphere on the left side), and (d) bottom view (left hemisphere on the right side) (*p* < 0.05, FWE corrected on the cluster level). List of peaks can be found in Table 3.

**Figure 6:**
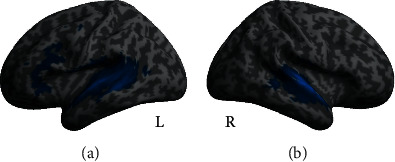
Neural response as measured by the BOLD response for the main effect of the listening condition. Peak activation sites include the left and right superior temporal gyri under easy compared to difficult listening conditions (blue). There are no significant peaks when comparing difficult to easy listening conditions. (a) Left hemisphere view and (b) right hemisphere view (*p* < 0.05, FWE corrected on the cluster level). List of peaks can be found in Table 3.

To investigate brain responses elicited during task performance, we computed a full factorial model with the factors group (hard of hearing vs. normal hearing), memory load (high vs. low), and listening condition (easy vs. difficult). The results are listed in Table 3. There were significant differences between the memory load conditions (main effect of memory load, Figure 5). To investigate this main effect, two directed contrasts were performed. BOLD response in the left supramarginal gyrus, left middle frontal gyrus, right cerebellum, and left supplementary motor cortex reflected increased activity under high memory load while responses in the left and right lingual gyri and in the left and right middle occipital gyri reflected increased BOLD activity under low memory load. We also found differences between listening conditions (main effect of listening condition, Figure 6). Follow-up contrasts revealed that all differences were due to activity increases in the easy listening condition. Hearing loss did not impact BOLD activity, nor did it interact with memory load or listening condition (no effect of the factor group, nor any interaction between any factors nor factor levels). Note that our contrasts for high memory load and difficult listening conditions within the regions of interest specified by the working memory and auditory effort masks revealed no significant results either.

A regression analysis modelling the sample as one group with hearing loss as measured by the mean PTA in the higher frequencies as a regressor also showed no significant association of BOLD response and hearing impairment.

We computed the beta values for all participants for the middle frontal gyrus for the contrast high versus low memory load (peak at *x* = −44, *y* = 24, and *z* = 30) and for the superior temporal gyrus for the easy versus difficult listening condition (peak at *x* = 62, *y* = −8, and *z* = 0). The Bayesian independent samples *T*-test with beta values showed evidence for the null hypothesis in all brain regions (all BF10 < 1), i.e., no difference between hard of hearing and normal-hearing participants in BOLD responses (see Table 4).

## 4. Discussion

This fMRI study is aimed at investigating sentence processing under increased demands on working memory and listening in participants with age-related hearing loss compared to an age-matched normal-hearing control group. This is the first study investigating verbal working memory in the context of a sentence processing task in hard-of hearing participants. We employed a 2 × 2 × 2 design with group (hard of hearing or normal hearing), memory load (high or low), and listening condition (difficult or easy). We expected larger drops of performance in hard of hearing participants under either high memory load or difficult listening conditions compared to normal-hearing participants. On the neural level, we hypothesized that BOLD responses in hard of hearing compared to normal-hearing participants are (1) increased in frontal brain areas, including the cingulo-opercular network, the inferior frontal and premotor cortices, the insula, and the middle frontal gyrus, when listening condition is difficult, and (2) increased in working memory-related areas, especially dorsolateral prefrontal and superior parietal cortex, under high memory load condition.

Our results on the behavioral level indicated that participants' performance was influenced by the listening condition only under low memory load, leading to a better performance when listening conditions were easy. Additionally, only under easy listening conditions memory load influenced the performance, leading to a decrease when memory load was high. This effect was, however, similar in hard of hearing and normal-hearing participants. On the neural level, participants showed increased BOLD responses in working memory areas in high compared to low memory load conditions and in auditory regions in easy compared to the difficult listening conditions. We found no significant differences in BOLD responses between the groups, and there were no significant interactions between any factors or factor levels.

### 4.1. Impact of Listening Condition and Memory Load on the Behavioral Level

The behavioral data provide evidence that challenges on memory load or listening impair task performance and interact with each other. Participants performed worse under high load as well as under difficult listening conditions. When investigating the interaction of memory load, listening condition, and group, there was a significant interaction effect of listening condition and memory load, but no interaction with the factor group. In both groups, the listening condition had an impact under low but not high memory load. Similarly, memory load modulated performance only under easy listening conditions. In other words, only when either memory load, listening condition, or both were difficult, performance was significantly worse. This interaction suggests that regardless of group, under more difficult conditions, cognitive resources are bound by either the stimulus perception or the solving of the task, which leads to a decline in performance. This interaction was significant for all participants; therefore, normal-hearing older adults are affected to the same extent as hard of hearing individuals by an increased working memory load or difficult listening condition. These were surprising findings, since we expected that hard of hearing participants in contrast to their normal-hearing peers would show higher drops in performance between high and low memory load conditions when listening was difficult as well as between easy and difficult listening conditions when memory load was high. An explanation for the lacking group effect might be that the difficult listening situation within the MRI resembles listening conditions to which the hard of hearing participants have already adapted to due to their decreased auditory input. This may explain why the sound level necessary to obtain 80% SRT during scanning did not differ significantly between the groups, which was also the case in our previous study [15]. Hence, hard of hearing participants are already used to understanding speech-in-noise, which might have made it possible to obtain the same level of performance as normal-hearing participants. Also, subjective difficulty ratings did not differ between groups, but revealed that the difficult listening condition was perceived as more difficult in both groups. Note that in contrast to the reduction in accuracy under high memory load, this high memory load condition was not judged as more difficult.

To sum up, our results support the theory that listening to speech in noise binds resources that could be used for other tasks [7, 34]. Hence, either a more complex cognitive task or perceptual difficulties can decrease performance. It is therefore important to take the memory load of any task into consideration (see [5, 8]).

### 4.2. Differences in Neural Responses between High and Low Memory Load Conditions

To investigate differences in BOLD responses, we performed a full factorial model with the factors group (hard of hearing vs. normal-hearing), memory load (low vs. high), and listening condition (easy vs. difficult). When investigating the effect of memory load, we found increased responses in several frontal and parietal areas. Areas with significantly increased BOLD responses under high compared to low memory load were found in the left middle frontal gyrus, the left supplementary motor cortex, the left supramarginal gyrus, and in the right cerebellum. The left middle frontal gyrus is a brain region that shows increased activity when more items need to be maintained in working memory [35, 36] and has also been associated with word production [37]. Furthermore, it is part of a frontoparietal network involved in cognitive control and attention guiding processes [38]. The left supramarginal gyrus is involved in word processing and phonological decision-making [39, 40]. The supplementary motor cortex also plays a role in working memory and has been associated with rehearsal of articulation. In addition, it belongs to the cingulo-opercular network which has shown increased functional coupling to the auditory cortex in age-related hearing loss [41, 42]. Hence, the activated areas that show increased neural recruitment in high compared to low memory load in all participants are responsible for a variety of functions including working memory, rehearsal, semantic, and word processing. Even though that neural activity in these regions has been related to effortful listening [13, 14, 17, 34, 43], we found no evidence for differential responses in hard of hearing participants. This suggests that our participants, regardless of hearing abilities, responded with similarly increased BOLD responses in speech processing areas to higher demands on working memory. This increased BOLD response may have facilitated solving the task. Comparing low to high memory load conditions revealed significantly higher BOLD responses in the middle occipital and lingual gyri. These areas are related to processing written speech [44, 45]. In the low memory load condition, participants had to read a longer question than in the high memory load condition (“Was the word x in the last sentence?” vs. “Same noun?”), which may explain the additional activity in visual brain regions.

These results suggest that neural activity as measured by the BOLD response of effortful speech perception and cognitive control due to higher demands on verbal working memory is increased, which is surprising as participants did not rate the high memory load condition as significantly more difficult than the low memory load condition. This highlights the importance of using neuroimaging measures of cognitive effort that do not rely on self-report to gain insight on listening in noise in the elderly, especially regarding age-related hearing loss. We conclude that participants responded to higher working memory load with increased BOLD response amplitudes in areas that aid speech perception to meet the increased demands of the task.

### 4.3. Differences in Neural Responses between Easy and Difficult Listening Conditions

Our fMRI data also showed a main effect of listening condition. Increased BOLD responses were rooted in the contrast of easy compared to difficult listening condition and comprised the left and right superior temporal gyrus. These areas are associated with speech perception and production [46, 47]. Hence, the results show that the increased sound pressure level of stimulation in the easy listening condition is reflected on a neural level as measured by the BOLD response. Contradictory to our expectations, we did not find any increased BOLD responses when comparing difficult to easy listening conditions in nonauditory areas. The two listening conditions may not have been distinct enough from each other to prompt the recruitment of nonauditory areas, and therefore, no significant change in BOLD response in frontal brain areas was obtained. Additionally, there are limited task design options for auditory fMRI studies. Results may be skewed as the MRI scanner noise may affect the hearing abilities of normal-hearing and hard of hearing participants differently and may further increase task difficulty. For further studies, the task might benefit from a sparse-sampling design, which would provide the opportunity to not only vary the listening condition but also influence the amount and type of noise instead of just using the scanner noise [48]. In our study, we used a sentence-based memory task as we wanted to mimic the experience of naturalistic speech. Exploring different cognitive tasks other than the sentence-based task we used or other forms of increased cognitive load (e.g., by an interfering dual task such as in Heinrich et al. [49]) may be useful in future studies.

### 4.4. No Significant Differences in Neural Responses between Hard of Hearing and Normal-Hearing Participants

We found no significant differences between hard of hearing and normal-hearing participants in brain activation patterns during a verbal working memory task. The additional Bayesian analysis provided evidence for the null hypothesis as well. To rule out noisy data as an explanation for our null effect, we calculated the SNR of the measurement using the MRIQC tool [50]. The SNRs in our sample range from 3.98 to 5.42 and are comparable to a younger sample measured with the same scanner and head coil and similar measurement parameters [51].

An explanation for the absence of group effect may be that the verbal working memory task generally was rather difficult, even in the easier conditions, and therefore was not sensitive enough to distinguish between normal-hearing and hard of hearing participants. A study by Cardin et al. [52] found less recruitment of frontal areas in deaf participants, who, in turn, relied more on superior temporal cortex during linguistic and nonlinguistic working memory tasks. The authors suggest that the profound hearing loss leads to a cortical reorganization related to working memory. Our mild to moderate hard of hearing sample may still retain enough hearing abilities or have not been impaired long enough to show such cortical reorganization. This may explain why they do not differ significantly from our normal-hearing group. These findings are in line with our prior study. Using a visual working memory paradigm, we previously found no evidence for neural differences between normal-hearing and hard of hearing participants either [53]. We now add evidence that the neural representation of auditory speech processing even seems intact in mild to moderate stages of hearing loss when a challenging verbal working memory task is used. In a more severely impaired sample, group differences may become more prominent as neural changes associated with presbyacusis are often found in more advanced stages of impairment [54, 55]. Hence, we suggest that with the increasing decline of hearing abilities, compensatory mechanisms may be necessary when listening conditions become too difficult [7].

## 5. Conclusion

This is the first study that provides results from a verbal working memory task that uses real-life, full-sentence stimuli in a sample of mild to moderate hard of hearing participants. Even though using sentences drastically increases task difficulty, we believe that it can provide valuable insights into a more natural way of language processing and mimic cognitive challenges that hard of hearing elderly often experience in daily life. There was a significant interaction of listening condition and memory load, whereby performance was only influenced by the listening condition under low memory load conditions, and similarly by memory load only under easy listening conditions. We found increased BOLD responses in high compared to low memory load conditions as well as to easy compared to difficult listening conditions. Under high memory load, frontoparietal regions were recruited regardless of hearing impairment; however, there was no additional recruitment of these regions under difficult listening conditions nor an interaction of memory load and listening condition. Increased BOLD responses in temporal regions in the easy listening condition are likely due to the loudness of the stimuli. We conclude that memory load and listening condition interacted on a behavioral level, whereas our task-imposed challenges interacted with verbal working memory, but not listening effort, on the neural level as measured by the BOLD response. The absence of group differences may indicate that the mild to moderate stage of hearing-loss may not yet be advanced enough to elicit significantly different response patterns from normal-hearing participants. BOLD responses for the different memory and listening conditions may only be altered in severe stages or longer durations of age-related hearing loss.

## Figures and Tables

**Table 1:** Characterization of participant groups.

	Normal hearing		Hard of hearing		*p*
	Mean	SD	Mean	SD	
Age	63.68	6.13	64.9	5.67	0.423
Handedness	88.51	17.06	79.34	26.67	0.211
GDS	1.16	1.38	1	1.03	0.687
CTMT Trail 5–Trail 1	35.11	30.17	34.30	21.21	0.923
MoCa	25.47	2.57	26.45	3.08	0.291
Stroop interference	62.89	7.01	60.85	5.31	0.310
WST	33.42	2.77	31.55	2.87	0.046∗
IED pre ED errors	8.63	4.95	6.89	1.88	0.161
IED ED errors	6.26	5.99	8.05	4.65	0.310

Note. CTMT: Comprehensive Trail Making Test (in seconds); MoCa: Montreal Cognitive Assessment; WST: verbal intelligence test; IED: intra-extradimensional set shift task; SD: standard deviation. ∗ denotes significance at *p* < 0.05.

**Table 2:** Hearing and speech tests.

	Normal hearing		Hard of hearing	
	Mean	SD	Mean	SD	*p*
High-frequency hearing loss (dB)	16.84	6.29	41.38	8.88	<0.001∗
OLSA (SNR)	-9.08	1.24	-7.83	1.64	0.011∗
OLSA (dB)	68.27	6.19	71.02	6.53	0.186
Subjective listening effort	2.72	1.58	2.98	1.12	0.550
ACALES low effort (SNR)	10.71	4.14	7.40	5.31	0.035∗
ACALES medium effort (SNR)	2.82	3.23	0.21	3.29	0.016∗
ACALES high effort (SNR)	-5.02	2.37	-6.63	2.94	0.067

Note: OLSA SNR: Oldenburger Sentence test performed outside the scanner; OLSA dB: Oldenburger Sentence test performed inside the scanner; ACALES: adaptive categorical listening effort scaling. ∗ denotes significance at *p* < 0.05.

**Table 3:** Peaks of neural response for the main effects in the verbal working memory task.

Main effect	Contrast	Peak (*x*, *y*, *z*)	*T*	Cluster	Brain region
Memory load	Low > high	16 -76 -4	8.93	1564	Right lingual gyrus
		-10 -82 -2	7.23	1144	Left lingual gyrus
		32 -82 16	6.32	793	Right middle occipital gyrus
		-26 -90 18	6.02	803	Left middle occipital gyrus

	High > low	-44 -48 44	5.95	1459	Left supramarginal gyrus
		-44 24 30	4.96	899	Left middle frontal gyrus
		38 -66 -34	4.50	324	Right cerebellum
		-8 26 46	4.28	366	Left supplementary motor cortex

Listening condition	Easy > difficult	62 -8 0	9.72	6809	Right superior temporal gyrus
		-60 -16 6	9.03	6056	Left superior temporal gyrus
	Difficult > easy	n.s.			

Group		n.s.			

Note: the specific contrasts of high > low memory load, low > high memory load, and easy > difficult sound encompass all areas reported in the main effects. (*p* < 0.05, FWE corrected on the cluster level). n.s.: not significant.

**Table 4:** Bayes null-hypothesis testing using independent samples *T*-tests for beta values at peaks for the verbal working memory task.

Contrast	Peak (*x*, *y*, *z*)	BF_10_	Error in %
Memory load high > low	-44 24 30	0.479	0.005
Listening condition easy > difficult	62 -8 0	0.367	0.004

Note: values smaller <1 indicate evidence for the null hypothesis.

## Data Availability

This study was preregistered on http://AsPredicted.com/ on the 27th of March 2019 and has the document number 21386. The fMRI data used to support the findings of this study have been deposited under https://osf.io/rs62e/, DOI: http://10.17605/osf.io/rs62e as well as an example trial of the verbal working memory task. This article has been published as a preprint: Pauquet, J., Thiel, C., Mathys, C., & Rosemann, S. (2020, May 6). Neural Representation of Auditory Speech Processing in Age-Related Hearing Impairment DOI: http://10.31219/osf.io/bpgr6.

## Supplementary Materials

Supplementary materials Supplementary Table 1: average ratings of the different subscales of the ACALES test for normal-hearing and hard of hearing participants. Supplementary Table 2: mean performance in the verbal working memory task.
