# Supplementary material for: Relationship between Memory Load and Listening Demands in Age-Related Hearing Impairment
Source: Neural Plast. 2021 Jun 4;2021:8840452. doi: 10.1155/2021/8840452 (PMC8195652; doi:10.1155/2021/8840452)
Supplement: Supplementary materials — Supplementary Table 1: average ratings of the different subscales of the ACALES test for normal-hearing and hard of hearing participants. Supplementary Table 2: mean performance in the verbal working memory task. [file 8840452.f1.docx]

**Supplement**

**Supplementary Table 1.** Scores from the Adaptive Categorical Listening Effort Scaling test.

| Rating | Hard of hearing | | Normal-hearing | | |  | |
| --- | --- | --- | --- | --- | --- | --- | --- |
|  | Mean | SD | | Mean | SD | | p |
| 1 no effort | 10.71 | 4.14 | | 7.40 | 5.31 | | .035* |
| 2 | 9.39 | 3.91 | | 6.19 | 4.88 | | .029* |
| 3 very little effort | 8.08 | 3.72 | | 4.98 | 4.49 | | .024* |
| 4 | 6.76 | 3.57 | | 3.78 | 4.13 | | .021* |
| 5 little effort | 5.45 | 3.47 | | 2.57 | 3.80 | | .018* |
| 6 | 4.13 | 3.38 | | 1.38 | 3.52 | | .017* |
| 7 moderate effort | 2.82 | 3.23 | | 0.21 | 3.29 | | .016* |
| 8 | 1.51 | 3.02 | | -0.95 | 3.09 | | .016* |
| 9 considerable effort | 0.21 | 2.75 | | -2.11 | 2.93 | | .015* |
| 10 | -1-11 | 2.51 | | -3.23 | 2.83 | | .017* |
| 11 very much effort | -2.41 | 2.34 | | -4.37 | 2.81 | | .023* |
| 12 | -3.72 | 2.31 | | -5.51 | 2.84 | | .037* |
| 13 extreme effort | -5.02 | 2.37 | | -6.63 | 2.94 | | .067 |

*Note.* Only every second level has a name. Scores are signal-to-noise ratios. * denotes significance at *p* < .05

**Supplementary Table 2**. Performance in the verbal working memory task.

|  | Normal-hearing | | Hard of hearing | | |  |
| --- | --- | --- | --- | --- | --- | --- |
|  | Mean | SD | | Mean | SD | |
| low memory load / easy listening | | | | | | |
| performance (%) | 77.96 | 16.71 | | 80 | 11.92 | |
| difficulty rating | 1.89 | 0.93 | | 2.2 | 0.83 | |
| low memory load / difficult listening |  |  | |  |  | |
| performance (%) | 61.18 | 11.89 | | 70.62 | 11.48 | |
| difficulty rating | 3.36 | 0.76 | | 3.4 | 0.82 | |
| high memory load / easy listening |  |  | |  |  | |
| performance (%) | 65.78 | 16.58 | | 67.18 | 13.58 | |
| difficulty rating | 2.21 | 0.91 | | 2.4 | 0.88 | |
| high memory load / difficult listening |  |  | |  |  | |
| performance (%) | 64.81 | 16.43 | | 64.68 | 14.93 | |
| difficulty rating | 3.57 | 0.69 | | 3.25 | 0.91 | |

*Note*. * denotes significance at p <.05.
